# *Bacillus thuringiensis* B1(2015b) is a Gram-Positive Bacteria Able to Degrade Naproxen and Ibuprofen

**DOI:** 10.1007/s11270-016-2893-0

**Published:** 2016-05-25

**Authors:** Ariel Marchlewicz, Dorota Domaradzka, Urszula Guzik, Danuta Wojcieszyńska

**Affiliations:** Department of Biochemistry, Faculty of Biology and Environmental Protection, University of Silesia in Katowice, Jagiellońska 28, 40-032 Katowice, Poland

**Keywords:** *Bacillus*, Naproxen, Ibuprofen, Biodegradation

## Abstract

A Gram-positive bacterium, designated as strain B1(2015b), was isolated from the soil of the chemical factory “Organika-Azot” in Jaworzno, Poland. On the basis of 16S rRNA gene sequence analysis, the isolated strain was classified as a *Bacillus thuringiensis* species. Strain B1(2015b) is able to degrade ibuprofen and naproxen, however, these compounds are not sufficient carbon sources for this strain. In the presence of glucose, *Bacillus thuringiensis* B1(2015b) degrades ibuprofen and naproxen with higher efficiency. Twenty milligrams per liter of ibuprofen was degraded within 6 days and 6 mg l^−1^ of naproxen was removed within 35 days. Simultaneously, the growth of the bacterial culture was observed. The obtained results suggest that *Bacillus thuringiensis* B1(2015b) appears to be a powerful and useful tool in the bioremediation of non-steroidal anti-inflammatory drugs-contaminated environment.

## Introduction

Non-steroidal anti-inflammatory drugs (NSAIDs) enter the environment as a result of pharmaceutical industry activity and the improper disposal of unused or expired drugs, waste generated in hospitals and stock-raising farms (Wu et al. 2012). In recent years, an increasing intake of the over-the-counter drugs such as ibuprofen, naproxen, paracetamol, ketoprofen, diclofenac, and acetylsalicylate has been observed (Wojcieszyńska et al. 2014; Domaradzka et al. 2015). Ibuprofen is the third most highly consumed pharmaceutical in the world (Murdoch and Hay 2015). For example, sale of ibuprofen in Poland and Germany reached 58 tonnes in 2000 and 345 tonnes in 2001, respectively (Nikolaou et al. 2007; Styszko et al. 2010). In 2000, 35 tonnes of naproxen were consumed in England (Nikolaou et al. 2007). This drug belongs to the most frequently detected pharmaceutical in the aquatic environment (Grenni et al. 2013). Although ibuprofen and naproxen are detected in surface water, ground water or even drinking water at a concentration within the ng l^−1^ to μg l^−1^ range, they may accumulate in aquatic organisms (Li et al. 2015a, b; Jeffries et al. 2015). The concentration of ibuprofen determined in the wild fish plasma and bile samples was 100 to 1000-fold higher than in water (Jeffries et al. 2015). It was also shown that ibuprofen, as a non-selective cyclooxygenase inhibitor, exhibits ecotoxic effects in fish mainly through the endocrine disruption (Yu et al. 2006). In contrast, photoderivatives of naproxen tends to be ecotoxic in acute and chronic conditions (Marotta et al. 2013).

In the environment, naproxen and ibuprofen may undergo sorption, desorption, biotransformation, and abiotic transformation (Lahti and Oikari 2011). The physico-chemical transformations lead to the formation of more toxic intermediates (Marotta et al. 2013; Li et al. 2015a). Therefore, bioremediation processes are an attractive alternative to these methods. Bioremediation strategies are cost-effective and enable the mineralization of NSAIDs into the safer products (Ahmed et al. 2001). However, these pharmaceuticals are difficult to biodegrade, and the efficiency of these processes is not satisfactory (Rodriguez-Rodriguez et al. 2010; Li et al. 2015b). The activated sludge process is used to treat the wastewaters containing non-steroidal anti-inflammatory drugs, but the removal of these drugs has been found to be incomplete (Rodarte-Morales et al. 2011). There is a need to isolate microorganisms with a high capacity for non-steroidal anti-inflammatory drug degradation. However, until now, only a few pure bacterial strains have been described to be able to degrade ibuprofen or naproxen. Murdoch and Hay (2005, 2015) described *Variovorax* Ibu-1 and *Sphingomonas* Ibu-2 engaged in the degradation of ibuprofen. Probably, degradation of ibuprofen by these strains occurs through catechol derivatives and the *meta* ring-fission (Murdoch and Hay 2005, 2015). During the degradation of ibuprofen by lignolytic bacteria *Nocardia* sp. NRRL 5646 formation of two metabolites, ibuprofenol and ibuprofenol acetate was observed (Chen and Rosazza 1994). Only one bacterial strain able to degrade naproxen was described. *Stenotrophomonas maltophilia* KB2 degrades this drug through the hydroxylation of the derivative of naproxen to hydroxyquinol, which is then cleaved by hydroxyquinol 1,2-dioxygenase (Wojcieszyńska et al. 2014).

Due to poor knowledge about the metabolism of the non-steroidal anti-inflammatory drugs in the environment, it is necessary to search for new pure bacterial strains that are able to degrade these compounds. In this study, the isolation and characterization of a Gram-positive *Bacillus thuringiensis* B1(2015b), which exhibits the ability to degrade naproxen and ibuprofen, has been reported for the first time.

## Materials and Methods

### Isolation of Pharmaceuticals Degrading Bacterium

Non-steroidal anti-inflammatory drugs-degrading strain was isolated from the soil of the chemical factory “Organika-Azot” in Jaworzno, Poland, using the classical enrichment technique with naproxen as a selection factor. The mixed microbial population from the soil was introduced to 0.85 % NaCl solution and shook at 30 °C in an aeration chamber. After 3 h, 1 ml samples were serially diluted from 10^−1^ to 10^−3^ with saline and spread onto the agar plates containing mineral salts medium (Na_2_HPO_4_ 12H_2_O 3.78 g; KH_2_PO_4_ 0.5 g; NH_4_Cl 5 g; MgSO_4_·7H_2_O; per liter of distilled water) with 6 mg l^−1^ naproxen to obtain pure cultures. The agar plates were incubated at 30 °C for 24 h and single colonies were isolated and transferred to the nutrient agar plates to test their purity. Single colonies showing different morphological characteristics were proliferated in a nutrient broth medium (at 30 °C on a rotary shaker at 130 rpm), harvested by centrifugation (5,000×*g* at 4 °C for 15 min) and washed with fresh sterile mineral salts medium. In order to verify, which strain is able to degrade naproxen and ibuprofen, cultures in a 250-ml flask containing 100 ml of a sterile mineral salts medium supplemented with 6 mg l^−1^ of naproxen or 20 mg l^−1^ of ibuprofen were inoculated with previously prepared cells to the final optical density of about 0.8–1.0 in absorbance scale at *λ* = 600 nm. The cultures were incubated with shaking at 130 rpm at 30 °C, and samples were drawn at regular intervals to monitor growth and naproxen or ibuprofen degradation. The strain able to degrade both naproxen and ibuprofen was kept as a freezing bacterial stock.

### Morphological, Physiological, and Biochemical Characterization of the Isolated Strain

The isolated strain was phenotypically and biochemically characterized using standard techniques (Gram staining, colony shape, size, and color on nutrient agar plate, etc.), according to Bergey’s Manual of Determinative Bacteriology (Holt et al. 1994). Additional biochemical and physiological characteristics were determined using the API Coryne system (BioMerieux, Lyon, France). Isolation of fatty acids was performed according to Sasser (1990). Analysis of FAMEs was performed using an HP 5890 gas chromatograph (Hewlett Packard, Rolling Meadows, IL, USA) equipped with an HP 25 m × 0.2 mm cross-linked methyl-silicone capillary column. The initial oven temperature was 170 °C, increased 5 °C min ^−1^ to 260 °C, the increased 40 °C min^−1^ and held constant at 320 °C for 1.5 min. Helium was used as the carrier gas. Fatty acid methyl esters (FAMEs) were identified with Sherlock software (TSBA library, version 3.9. Microbial ID, Newark, NJ, USA) based on the actual calibration retention times run prior to sample analysis.

### Analytical Methods

The concentration of non-steroidal anti-inflammatory drugs: ibuprofen, naproxen, paracetamol, and diclofenac (introduced as pharmaceutical substances) were determined with the HPLC technique using Merck Hitachi HPLC reversed-phase chromatograph equipped with a column Ascentis Express ® C18 HPLC Column (100 × 4.6 mm), pre-column Opti-Solw ® EXP, and UV/VIS DAD detector. The mobile phase consisted of acetonitrile and 1 % acetic acid (50:50 *v/v* for naproxen, vanillic acid, protocatechuic acid, benzoic acid, and 4-hydroxybenzoic acid assay and 5:95 *v/v* for ibuprofen assay) at a flow rate of 1 ml/min. The mobile phase consisted of acetonitrile, 1 % acetic acid, and methanol (50:30:20 *v/v/v* for diclofenac assay and 20:60:20 *v/v/v* for phenol assay) or methanol and 1 % acetic acid (5:95 *v/v*) for paracetamol assay at a flow rate of 1 ml/min. The detection wavelength was set at 260 nm (naproxen, vanillic acid, protocatechuic acid, benzoic acid, and 4-hydroxybenzoic acid), 240 nm (ibuprofen and paracetamol), 272 nm (phenol), and 276 nm (diclofenac) (Wojcieszyńska et al. 2014). Ibuprofen, naproxen, paracetamol, diclofenac, phenol, vanillic acid, protocatechuic acid, benzoic acid, and 4-hydroxybenzoic acid were identified by comparing the HPLC retention times and UV-visible spectra with those of the external standards. The concentration of glucose in the culture supernatant was determined using colorimetric anthrone method (Gerhardt et al. 1994). The concentration of salicylic acid was determined by the method with iron(III) chloride (Poljudek-Fabini and Bejrih 1981).

### Naproxen and Ibuprofen Degradation Experiments

Strain B1(2015b) was routinely cultivated in the nutrient broth at 30 °C and 130 rpm for 24 h. After this, cells were harvested by centrifugation (5,000×*g* at 4 °C for 15 min), washed with a fresh sterile medium, and used as inoculum.

Degradation of naproxen or ibuprofen in monosubstrate systems were performed in 500-ml Erlenmeyer flasks containing 250 ml of the mineral salts medium (Greń et al. 2010) inoculated with cells to a final optical density of about 0.8 at *λ* = 600 nm (OD600). Naproxen was added to obtain a final concentration of 6 mg l^−1^, and all cultures were incubated with shaking at 30 °C for 35 days. The chromatographic analyses of the culture fluid and measurements of the cultures growth were carried out every 7 days. For the studies on ibuprofen degradation, strain B1(2015b) was grown in a mineral salts medium supplemented with ibuprofen (concentration range 1–25 mg l^−1^). The residual ibuprofen concentration in the culture filtrates was assayed by liquid chromatography every 24 h.

For studies on the cometabolic degradation of naproxen or ibuprofen, 1 mg l^−1^ glucose was added. Cultures in 250 ml of sterile mineral salt medium supplemented with glucoses and 6 mg l^−1^ naproxen or 1–25 mg l^−1^ ibuprofen were inoculated with cells to a final optical density of about 0.1 at *λ* = 600 nm (OD600) and incubated at 30 °C with shaking at 130 rpm. If the complete degradation of the suitable growth substrate was observed, a successive dose of glucose was introduced and the culture was left for incubation until it reached OD_600_ = 1.0. All cultures were grown in triplicates.

Additionally, control cultures (250 ml) for each drug were prepared: an uninoculated control consisted of the mineral salts medium only (abiotic degradation control), and a heat-killed control consisted of bacterial cells destroyed by autoclaving (adsorption onto biomass control). The optical density of the heat-killed control was the same as for the examined cultures.

### Phylogenetic Analysis

Bacterial DNA was isolated from the pure culture using the DNA commercial kit (GenElute Bacterial Genomic DNA Kit, Sigma-Aldrich). For 16S rRNA gene amplification, the bacteria-specific primers: 8 F 5′AGTTTGATCATCGCTCAG 3′ and 1492R 5′GGTTACCTTGTTACGACTT3′ were used. Amplification was carried out through a program consisting of initial denaturation at 94 °C for 300 s, three cycles at 94 °C for 45 s, 57 °C for 30 s, and 72 °C for 120 s; three cycles at 94 °C for 45 s, 56 °C for 30 s, and 72 °C for 120 s; three cycles at 94 °C for 45 s, 55 °C for 30 s, and 72 °C for 120 s; 26 cycles at 94 °C for 45 s, 53 °C for 30 s, and 72 °C for 120 s; and a final elongation step at 72 °C for 300 s. The nucleotide sequencing of the gene was done by using the Big Dye^R^ Terminator Cycle Sequencing Kit (Applied Biosystem) and AbiPrism®3100 Genetic Analyzer. The MegaBLAST program was used for homology searches with the standard default program. Multiple sequence alignments were performed and the neighbor-joining phylogenetic tree was constructed using CLC Sequence Viewer 7.0.2 program. The 16S rRNA gene sequence determined in this study has been deposited in the GeneBank database of NCBI under the accession number KP895873.1.

## Results and Discussion

### Isolation and Identification of Strain that Degrades Aromatic Compounds

Until now, only a few strains have been isolated and characterized as naproxen or ibuprofen degraders. The ability to degrade ibuprofen was shown for *Sphingomonas* sp. Ibu-2 and *Variovorax* Ibu-1 (Murdoch and Hay 2005, 2015) as well as for *Patulibacter* sp. strain I11 (Almeida et al. 2013). The only strain able to degrade naproxen is *Stenotrophomonas maltophilia* KB2, which degrades this compound under cometabolic conditions (Wojcieszyńska et al. 2014). However, a bacterial strain that would have shown the ability to degrade both ibuprofen and naproxen has not yet been described.

**Fig. 1:**
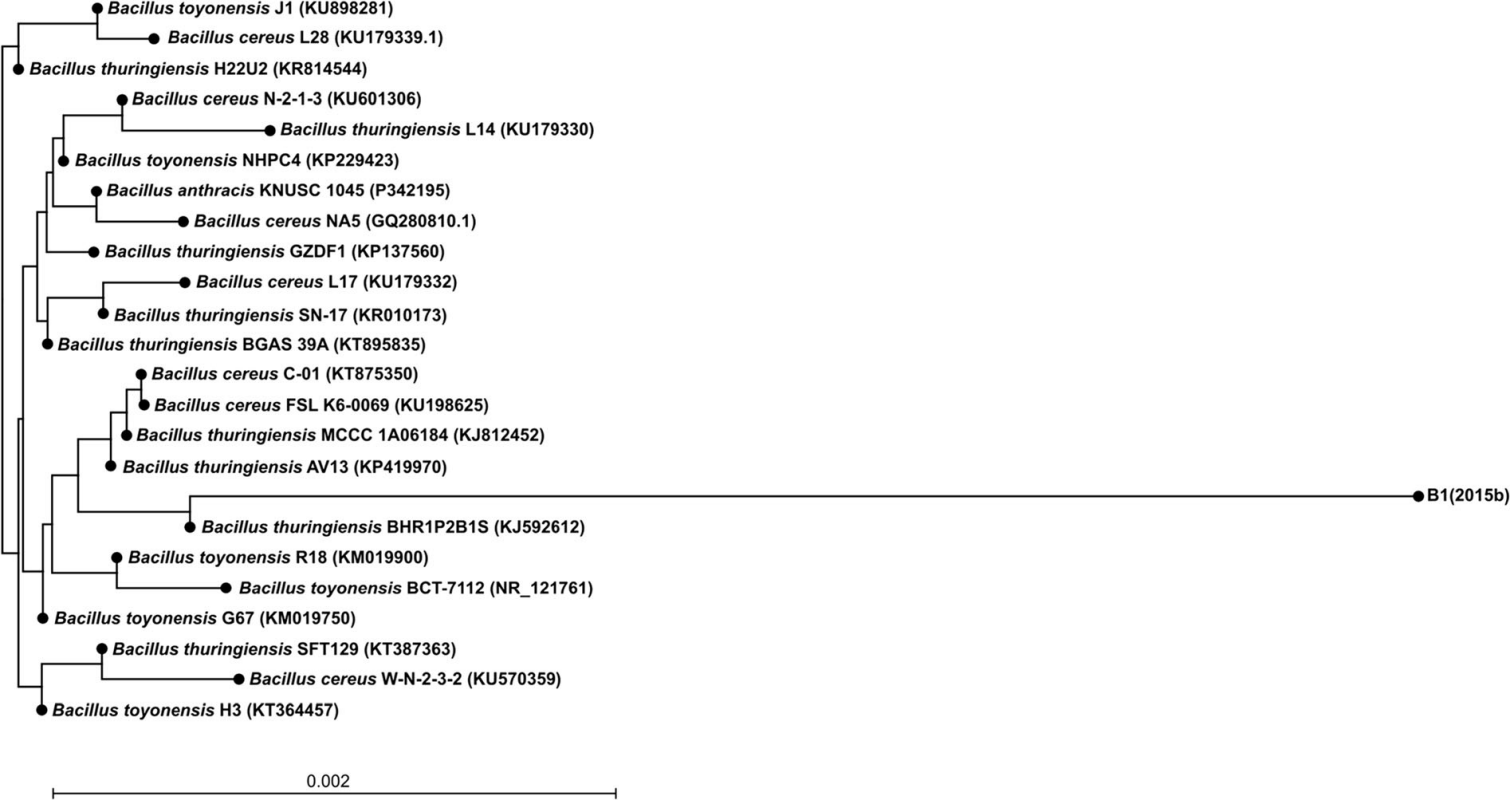
Neighbor-joining tree showing the phylogenetic position of the non-steroidal anti-inflammatory-degrading bacterium strain B1(2015b) and selected and related species of the genus *Bacillus* based on partial 16S rRNA gene sequences. The GenBank accession number for each microorganism used in the analysis is shown in parentheses after the species name

The strain marked as B1(2015b), which was isolated from the soil of the chemical factory “Organika-Azot” in Jaworzno, Poland, is a Gram-positive rod-shaped bacterium able to utilize two of the five most commonly used non-steroidal anti-inflammatory drugs: ibuprofen and naproxen. In contrast, the strain is not capable of degrading salicylic acid, acetaminophen, and diclofenac. Moreover, this strain is able to use various aromatic compounds as a carbon and energy source: phenol, vanillic acid, protocatechuic acid, benzoic acid, and 4-hydroxybenzoic acid. Microbiological and biochemical characterization of the strain revealed that it is aerobic, oxidase, and catalase positive (Table 1). Colonies of strain B1(2015b) were found to be circular, smooth, convex, and cream-colored. The biochemical and physiological characteristics of strain B1(2015b) are summarized in Table 1. The analysis of the fatty acid profile showed significant contents of 18:0 *anteiso* and 16:0 and 18:0 fatty acids (Table 2). It is known that straight and branched chain fatty acids are biomarkers of Gram-positive bacteria (Piotrowska-Seget and Mrozik 2003). However, we also observed an unusual amount of 18:1 ω9c fatty acids. These results coincide with the results obtained by Li et al. (2010) who observed 30.96 % of 18:1 fatty acids in *Bacillus subtilis*. Partial sequence analysis of the 16S rRNA gene allows classifying the isolate with 98 % similarity as a member of the genus *Bacillus*. Comparison of the 16S rDNA gene sequence of the isolate with the 16S rDNA gene sequences of bacteria identified to a species level showed that selected strain belongs to the species *Bacillus thuringiensis* (Fig. 1). In accordance with these data, the isolate B1(2015b) was included in the genus *Bacillus* and named as *Bacillus thuringiensis* sp. B1(2015b).

**Table 1:** Differential phenotypic characteristics of strain B1(2015b)

Characteristic	Results
Growth in the absence of NaCl	+
Growth in the presence of 1.5 % (*w/v*) NaCl	+
Growth in the presence of 3 % (*w/v*) NaCl	+
Growth at 4 °C	+
Growth at 20 °C	+
Growth at 30 °C	+
Growth at 42 °C	−
Oxidase	+
Catalase	+
Hydrolysis of esculin	+
Hydrolysis of gelatin	−
Arginine dihydrolase	−
Urease	−
Indol production	−
Nitrate reduction	−
Pyrazinamidase	−
Pyrrolidonyl arylamidase	−
Alkaline phosphatase	+
β-Glucuronidase	−
β-Galactosidase	−
α-Glucosidase	+
N-acetyl-β-glucosaminidase	−
Assimilation of:	
Glucose	+
Arabinose	−
Mannose	+
Mannitol	+
Maltose	+
Gluconate	+
Caprate	−
Adipate	−
Malate	−
Citrate	−
Phenylacetate	+
N-acetyl-glucosamine	+
Fermentation of:	
Glucose	+
Ribose	−
Xylose	−
Mannitol	−
Maltose	−
Lactose	−
Saccharose	−
Glycogen	−

*+* positive reaction, *−* negative reaction

**Table 2:** Percentage of total fatty acid from *Bacillus thuringiensis* B1(2015b)

Fatty acids	% of total fatty acids
Saturated	
14:0	0.21 ± 0.011
16:0	9.09 ± 0.218
17:0	0.28 ± 0.025
18:0	4.58 ± 0.24
18:0 2OH	0.34 ± 0.02
18:0 *anteiso*	47.42 ± 0.26
19:0 *iso*	0.31 ± 0.05
20:0	0.19 ± 0.00
Unsaturated	
16:1 ω7c	0.19 ± 0.03
17:1 ω8c	0.16 ± 0.00
18:1 ω9c	37.46 ± 0.13
Sat./unsat. ratio	0.18

–OH indicates the position of hydroxyl group from the acid end

*ω* methyl end of fatty acid, *c cis* configuration of the double bound, *iso anteiso*-branched fatty acids

### Degradation of Ibuprofen and Naproxen by Strain B1(2015b)

It is the first report on Gram-positive bacterium that is able to degrade both naproxen and ibuprofen. The described strain was isolated from a post-industrial landfill site belonging to a chemical factory which has produced veterinary drugs, hygiene products, and pesticides since 1928. It caused contamination of the surrounding area by cyanides, heavy metals, and pesticides such as dieldrin, endrin, α-, β-, and γ-hexachlorocyclohexane, phenols, hexachlorobenzene, dichlorodiohenyltrichloroethane (DDT), dichlorodiphenyldichloroethylene (DDE), and dichlorodiphenyldichloroethane (DDD). These contaminations probably influence the adaptation of microflora in this area. That is why we could assume that it is a good source of microorganisms able to degrade different aromatic compounds. The bacterium isolated during this study was classified as a *Bacillus*. It is known that Gram-positive bacteria, including the *Bacillus* species, are tolerant to many toxic compounds such as phenols, policyclic aromatic hydrocarbons, heavy metals, and different organic solvents. The high tolerance to different factors is connected with the structure of cellular membranes, synthesis of the surface active agents, and specific enzymes (Satchanska et al. 2006; Trivedi et al. 2011; Solyanikova et al. 2014; Swaathy et al. 2014).

**Fig. 2:**
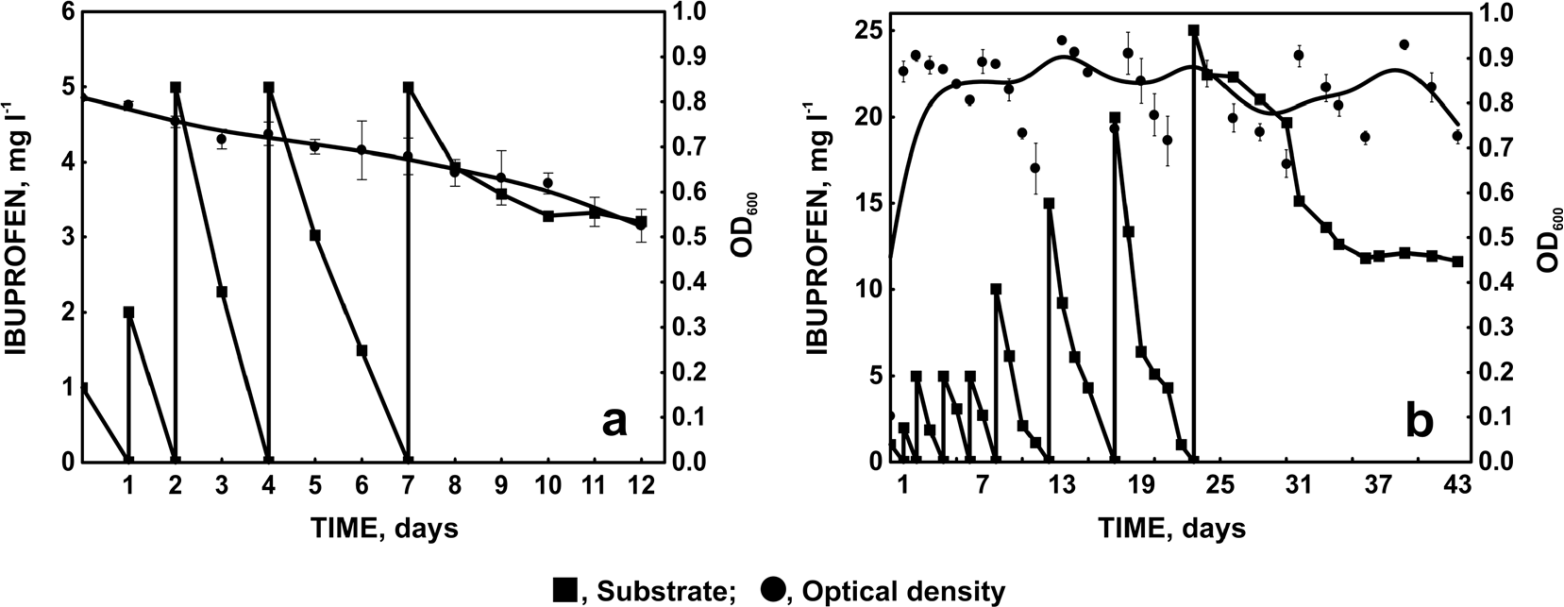
The adaptation of *Bacillus thuringiensis* B1(2015b) to increasing concentration of ibuprofen and changes of microbial biomass monitored as optical density at 600 (**a** without additional carbon source; **b** with 1 ml mg l^−1^ glucose as a simple carbon source)

**Fig. 3:**
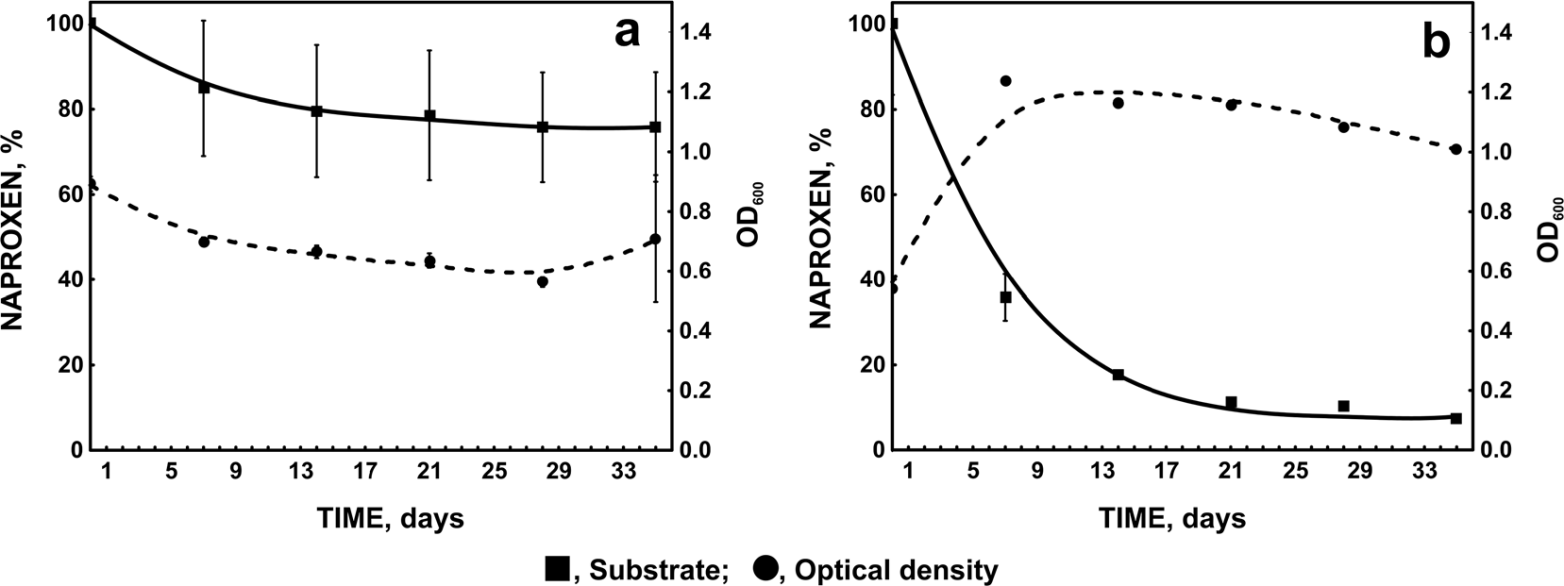
Degradation of 6 mg l^−1^ naproxen by strain B1(2015b) and changes of microbial biomass monitored as optical density at 600 nm (**a** without additional carbon source; **b** with 1 ml mg l^−1^ glucose as a simple carbon source)

The isolated strain, *Bacillus thuringiensis* B1(2015b), exhibits the ability to degrade 5 mg l^−1^ of ibuprofen within 2 days (Fig. 2a) and small amounts of naproxen (Fig. 3a). However, we observed a decrease in the optical density of bacterial cultures (Figs. 2a and 3a). It indicates that these compounds are not a sufficient carbon source. It is known that an additional carbon source may enhance the degradation capability of the strain by increasing the biomass (Zhong et al. 2007; Zhang et al. 2009). Quintana et al. (2005) observed the intensification of naproxen and ibuprofen degradation by active sludge in the presence of powdered milk used as a growth substrate. In this study, glucose was used as a carbon and energy source. It resulted in the significant improvement of NSAIDs’ degradation activity of the examined strain. Simultaneously, the decrease of the biomass was not observed. Under this condition, strain B1(2015b) was adapted to the degradation of 20 mg l^−1^ of ibuprofen within 6 days. However, 46.56 % of 25 mg/l of ibuprofen was degraded by this strain during 20 days (Fig. 2b).

Murdoch and Hay (2005, 2015) also described bacterial strains *Sphingomonas* Ibu-2 and *Variovorax* Ibu-1 able to degrade ibuprofen at high concentration. However, *Bacillus thuringiensis* B1(2015b) was additionally able to totally degrade 6 mg l^−1^ of naproxen within 35 days (Fig. 3b). Complete degradation of naproxen by a pure bacterial strain has not yet been observed. The described *Stenotrophomonas maltophilia* strain KB2 degraded 78 % of 6 mg l^−1^ of naproxen in the presence of glucose (Wojcieszyńska et al. 2014).

To conclude, bacterial strain isolated from the soil of the chemical factory “Organika-Azot” in Jaworzno, Poland, was identified as *Bacillus thuringiensis* B1(2015b). This strain showed the ability to degrade 20 mg l^−1^ of ibuprofen during 6 days and 6 mg l^−1^ of naproxen during 5 weeks under cometabolic condition. Because of these facts, *Bacillus thuringiensis* B1(2015b) may play a key role in the bioremediation of NSAIDs-contaminated environments.

## References

[CR1] Ahmed S, Javed MA, Tanvir S, Hameed A (2001). Isolation and characterization of a *Pseudomonas* strain that degrades 4-acetamidophenol and 4-aminophenol. Biodegradation.

[CR2] Almeida B, Kjeldal H, Lolas I, Knudsen AD, Carvalho G, Nielsen KL (2013). Quantitative proteomic analysis of ibuprofen-degrading *Patulibacter* sp. strain I11. Biodegradation.

[CR3] Chen Y, Rosazza JPN (1994). Microbial transformation of ibuprofen by a *Nocardia* species. Applied and Environmental Microbiology.

[CR4] Domaradzka D, Guzik U, Wojcieszyńska D (2015). Biodegradation and biotransformation of polycyclic non-steroidal anti-inflammatory drugs. Reviews in Environmental Science and Biotechnology.

[CR5] Gerhardt P, Murray RGE, Wood WA, Krieg NR (1994). Methods for general and molecular bacteriology.

[CR6] Greń I, Wojcieszyńska D, Guzik U, Perkosz M, Hupert-Kocurek K (2010). Enhanced biotransformation of mononitrophenols by *Stenotrophomonas maltophilia* KB2 in the presence of aromatic compounds of plant origin. World Journal of Microbiology and Biotechnology.

[CR7] Grenni P, Patrolecco L, Ademollo N, Tolomei A, Caracciolo AB (2013). Degradation of gemfibrozil and naproxen in a river water ecosystem. Microchemical Journal.

[CR8] Holt JG, Krieg NR, Sneath PHA, Staley JT, Williams ST (1994). Bergey’s manual of determinative bacteriology.

[CR9] Jeffries KM, Brander SM, Britton MT, Fangue NA, Connon RE (2015). Chronic exposure to low and high concentration of ibuprofen elicit different gene response patterns in a euryhaline fish. Environmental Science and Pollution Research.

[CR10] Lahti M, Oikari A (2011). Microbial transformation of pharmaceuticals naproxen, bisprolol, and diclofenac in aerobic and anaerobic environments. Archives of Environmental Contamination and Toxicology.

[CR11] Li Y, Wu S, Wang L, Li Y, Shi F, Wang X (2010). Differentiation of bacteria using fatty acid profiles from gas chromatography—tandem mass spectrometry. Journal of the Science of Food and Agriculture.

[CR12] Li FH, Yao K, Lv WY, Liu GG, Chen P, Huang HP, Kang YP (2015). Photodegradation of ibuprofen under UV–vis irradiation: mechanism and toxicity of photolysis products. Bulletin of Environmental Contamination and Toxicology.

[CR13] Li X, Toledo RA, Wang S, Shim H (2015). Removal of carbamazepine and naproxen by immobilized *Phanerochaete chrysosporium* under non-sterile condition. New Biotechnology.

[CR14] Marotta R, Spasiano D, Di Somma I, Andreozzi A (2013). Photodegradation of naproxen and its photoproducts in aqueous solution at 254 nm: a kinetic investigation. Water Research.

[CR15] Murdoch RW, Hay AG (2005). Formation of catechols *via* removal of acid side chains from ibuprofen and related aromatic acid. Applied and Environmental Microbiology.

[CR16] Murdoch RW, Hay AG (2015). The biotransformation of ibuprofen to trihydroxyibuprofen in activated sludge and by *Variovorax* Ibu-1. Biodegradation.

[CR17] Nikolaou A, Meric S, Fatta D (2007). Occurrence patterns of pharmaceuticals in water and wastewater environments. Analytical and Bioanalytical Chemistry.

[CR18] Piotrowska-Seget Z, Mrozik A (2003). Signature lipid biomarker (SLB) analysis in determining changes in community structure of soil microorganisms. Polish Journal of Environmental Studies.

[CR19] Poljudek-Fabini R, Bejrih T (1981). Organicheskij analiz.

[CR20] Quintana JB, Weiss S, Reemtsma T (2005). Pathways and metabolites of microbial degradation of selected acidic pharmaceutical and their occurrence in municipal wastewater treated by a membrane bioreactor. Water Research.

[CR21] Rodarte-Morales AI, Feijoo G, Moreira MT, Lema JM (2011). Degradation of selected pharmaceutical and personal care products (PPCPs) by white-rot fungi. World Journal of Microbiology and Biotechnology.

[CR22] Rodriguez-Rodriguez CE, Marco-Urrea E, Caminal G (2010). Degradation of naproxen and carbamazepine in spiked sludge by slurry and solid-phase *Trametes versicolor* systems. Bioresource Technology.

[CR23] Sasser, M. (1990). *Technical note* #, 101–110.

[CR24] Satchanska G, Topalova Y, Ivanov I, Golovinsky E (2006). Xenobiotic biotransformation potential of *Pseudomonas rhodesiae* KCM-R_5_ and *Bacillus subtilis* KCM-RG_5_, tolernat to heavy metals and phenol derivatives. Biotechnology & Biotechnological Equipment.

[CR25] Solyanikova IP, Robota IV, Mazur DM, Lebedev AY, Golovleva LA (2014). Application of *Bacillus* sp. strain VT-8 for decontamination of TNT-polluted sites. Microbiology.

[CR26] Styszko K, Sosnowska K, Wojtanowicz P, Gołaś J, Górecki J, Macherzyński M (2010). Sorption of ibuprofen on sediments from the Dobczyce (southern Poland) drinking water reservoir. Archives of Environmental Protection.

[CR27] Swaathy S, Kavitha V, Pravin AS, Mandal AB, Gnanamani A (2014). Microbial surfactant mediated degradation of anthracene in aqueous phase by marine *Bacillus licheniformis* MTCC5514. Biotechnology Reports.

[CR28] Trivedi N, Gupta V, Kumar M, Kumari P, Reddy CRK, Jha B (2011). Solvent tolerant marine bacterium *Bacillus aquimaris* secreting organic solvent stable alkaline cellulase. Chemosphere.

[CR29] Wojcieszyńska D, Domaradzka D, Hupert-Kocurek K, Guzik U (2014). Bacterial degradation of naproxen—undisclosed pollutant in the environment. Journal of Environmental Management.

[CR30] Wu S, Zhang L, Chen J (2012). Paracetamol in the environment and its degradation by microorganism. Applied Microbiology and Biotechnology.

[CR31] Yu JT, Bouwer EJ, Coelhan M (2006). Occurrence and biodegradability studies of selected pharmaceuticals and personal care products in sewage effluent. Agricultural Water Management.

[CR32] Zhang G, Yang X, Xie F, Chao Y, Qian S (2009). Cometabolic degradation of mono-chloro benzoic acids by *Rhodococcus* sp. R04 grown on organic carbon sources. World Journal of Microbiology and Biotechnology.

[CR33] Zhong Y, Luan T, Wang X, Lan CH, Tam NFY (2007). Influence of growth medium on cometabolic degradation of polycyclic aromatic hydrocarbons by *Sphingomonas* sp. strain PheB4. Applied Microbiology and Biotechnology.

